# Microbiota diversity of *Anopheles gambiae* in Bankeng, southern Cameroon, and its association with *Plasmodium falciparum* infection

**DOI:** 10.1128/msphere.00490-25

**Published:** 2025-12-05

**Authors:** Maurice Marcel Sandeu, Claudine Grâce Maffo Tatsinkou, Nsa Dada, Franklin Kouhoué Feukam, Flobert Njiokou, Grant L. Hughes, Charles S. Wondji

**Affiliations:** 1 Department of Medical Entomology, Centre for Research in Infectious Diseases (CRID), LSTM Research Unit 731240, Yaoundé, Cameroon; 2 Department of Microbiology and Infectious Diseases, School of Veterinary Medicine and Sciences, University of Ngaoundéré 198775, Ngaoundere, Cameroon; 3 Department of Animal Biology and Physiology, Faculty of Science, University of Yaoundé 107751, Yaoundé, Cameroon; 4 School of Life Sciences, Arizona State University 7864, Tempe, Arizona, USA; 5 Ecole Polytechnique, Palaiseau, France; 6 Departments of Vector Biology and Tropical Disease Biology, Centre for Neglected Tropical Diseases, Liverpool School of Tropical Medicine 9655, Liverpool, United Kingdom; 7 Department of Vector Biology, Liverpool School of Tropical Medicine 9655, Liverpool, United Kingdom; The University of Texas Medical Branch at Galveston, Galveston, Texas, USA

**Keywords:** malaria, *Anopheles gambiae*, microbiota, *Plasmodium falciaparum*, Cameroon

## Abstract

Malaria control requires the coordination of different strategies due to the lack of an effective vaccine and the emerging resistance of parasites to drugs and of vectors to insecticides. Therefore, efficient and environmentally safe alternative control strategies are still needed. In this study, we explored the composition of microbiota of the *Anopheles gambiae* and its variability in the presence of natural *Plasmodium* infection during the wet and dry seasons, in order to determine their potential as a novel vector control-based approach to fight malaria. An entomological survey of a collection of *An. gambiae* was conducted in Bankeng. Using 16S ribosomal RNA amplicon-based sequencing, we investigated the bacterial microbiota of mosquitoes naturally infected or uninfected with *Plasmodium falciparum*. A total of 120 mosquitoes were selected randomly corresponding to 60 mosquitoes per infection status. Overall, 99 bacterial taxa were detected across all the samples, with 97 of these shared between uninfected and infected. A total of two were unique to uninfected (*Acetobacteraceae*, *Enterococcus*), while no bacteria were unique to infected mosquitoes. However, there were significant differences in bacterial composition between both groups. Additionally, differential abundance revealed notable variations in microbiota composition, with 14 bacterial genera more abundant in uninfected mosquitoes and only two bacterial genera more abundant in the infected mosquitoes. Observed amplicon sequence variants and Shannon indices revealed a significant difference in bacterial diversity between infected (positive) and uninfected (negative) *An. gambiae* with higher diversity observed in negative samples during the wet season and in positive samples during the dry season. These findings highlight potential associations between certain bacterial taxa and infection status, suggesting they may be linked to susceptibility or resistance, although causality cannot be determined from this study. Ultimately, this baseline information provides a foundation for studies on the functions and interactions of the microbiota in natural populations of *Anopheles gambiae* and their susceptibility to natural *Plasmodium* infection.

**IMPORTANCE:**

Malaria control faces challenges due to the absence of an effective vaccine and growing resistance to drugs and insecticides, highlighting the need for alternative strategies. This study investigates the microbiota composition of *Anopheles gambiae* mosquitoes in Bankeng, Cameroon, and its association with natural *Plasmodium falciparum* infection. Using 16S rRNA amplicon sequencing, the bacterial communities of 120 mosquitoes—60 infected and 60 uninfected—were analyzed. A total of 99 bacterial taxa were identified, with 97 shared between both groups. Only two taxa (*Acetobacteraceae* and *Enterococcus*) were exclusive to uninfected mosquitoes, and none were unique to the infected group. Significant differences in microbiota composition were observed: 14 bacterial genera were more abundant in uninfected mosquitoes, while only two were enriched in infected ones. These findings suggest that specific bacteria may influence susceptibility to *Plasmodium* infection. This study provides foundational knowledge for exploring microbiota-based or paratransgenic strategies in malaria vector control.

## INTRODUCTION

Current malaria elimination efforts highlight the promising role of mosquito microbiota in disrupting the transmission of vector-borne diseases (1, 2). Mosquito microbiota are involved in many important biological processes, such as the production of the peritrophic matrix (3), the stimulation of basal immune activity in the gut, nutrition, digestion, development, and resistance to pathogens (4). The mosquito microbial composition is complex and depends on several factors such as the acquisition of environmental microbes, seasonality, and genetic parameters (5, 6). These variations of microbiota in *Anopheles* mosquitoes may indicate the variation in vector competence observed in different localities.

The ability of mosquitoes to transmit pathogens is influenced by the bacterial microbiota. Certain bacterial species can increase or reduce (7) vector susceptibility to pathogens and have the potential to alter vectorial capacity (8). Recent evidence also suggests that some members of these microbial communities can potentially interact with mosquito-borne pathogens both directly and indirectly. In *Anopheles gambiae*, *Asaia* was more abundant in *Plasmodium*-negative mosquitoes, implying a potential inhibitory effect. Conversely, in *An. coluzzii*, *Asaia* levels were higher in *Plasmodium*-infected individuals, with all infected samples containing *Asaia*, suggesting a possible role in facilitating infection (9). Additionally, another study revealed that different strains of *Serratia* bacteria can have varying effects on *Plasmodium* infections (10). Bai et al. identified two mosquitoes symbiotic *Serratia* strains *Serratia* Y1 and *Serratia* J1 from field-caught *An. sinensis* mosquitoes, with different contrasting effects on *Plasmodium berghei* development. The Serratia Y1 strain showed anti-*Plasmodium* activity, while Serratia J1 did not influence parasite development in the midgut of mosquitoes (11). While these studies examine specific bacterial taxa with distinct effects on particular host lines, our focus was on understanding how the overall microbiota interacts with *Plasmodium falciparum* in *Anopheles* mosquitoes.

Most studies investigating microbiota-pathogen interactions use antibiotic treatments to eliminate the microbiota and assess the impact of development success of *Plasmodium* (12, 13). Such treatments were provided via the incorporation of antibiotics in sugar meal at non-therapeutic concentrations but have been shown to cause dysbiosis rather than a complete elimination of the microbiota (14). Altering midgut bacteria in *Anopheles* mosquitoes through antibiotic treatments significantly influences the mosquito’s vectorial capacity, often enhancing *Plasmodium* development (12, 15, 16). These experiences provide evidence of the microbiota’s role in vector competence.

Despite extensive research on the mosquito microbiota, scientific understanding of the interactions between *Plasmodium* and the native microbial communities of *Anopheles gambiae* remains limited. In contrast, Hubert et al. reported greater microbiota diversity in *P. falciparum*-infected *An. gambiae* and *An. funestus* compared to non-infected mosquitoes without investigating microbiota variation across different seasons. These findings suggest that the microbiota may play a role in determining the mosquito’s ability to transmit pathogens (17). However, given the lack of comprehensive data on the specific microbiota of mosquitoes, particularly those with potential antagonistic effects on malarial parasites, primary studies to gather sufficient information are crucial in identifying bacteria that could negatively affect parasite development in vectors. To date, no comprehensive studies on the microbiota and *P. falciparum* in natural populations of *An. gambiae* from Cameroon have been reported.

The main purpose of this work is to investigate the changes in the microbial composition within the *An. gambiae* mosquito vector. The key research question is whether the overall abundance and diversity of *Anopheles* microbial communities change in the presence of a natural *P. falciparum* infection. Therefore, the aim of this study is to evaluate the composition and structure of the microbiota in *P. falciparum*-infected and non-infected *An. gambiae* in Bankeng. This research presents an exciting opportunity to advance our knowledge of the interactions between the *Anopheles*, microbiota, and *Plasmodium*.

## RESULTS

### Mosquito collection and *Plasmodium* infection rates by qPCR per season

A total of 975 mosquitoes were collected and morphologically identified as *Anopheles gambiae* s.l. in this locality during both seasons using morphological identification. The distribution of mosquito species differed significantly between the two seasons. More mosquitoes were collected in the rainy season compared to the dry season (dry season total = 220; rainy season total = 755; *P* = 0.004). To confirm species identity, molecular identification was conducted on all 220 specimens collected during the dry season and 334 randomly selected specimens from the rainy season collection. During both seasons, the collection was predominantly *An. gambiae* (*n* = 554, 99.5%), with only three *An. coluzzii* individuals. Consequently, upcoming research into *Plasmodium* detection and microbiota characterization will focus exclusively on *An. gambiae*.

**Fig 1:**
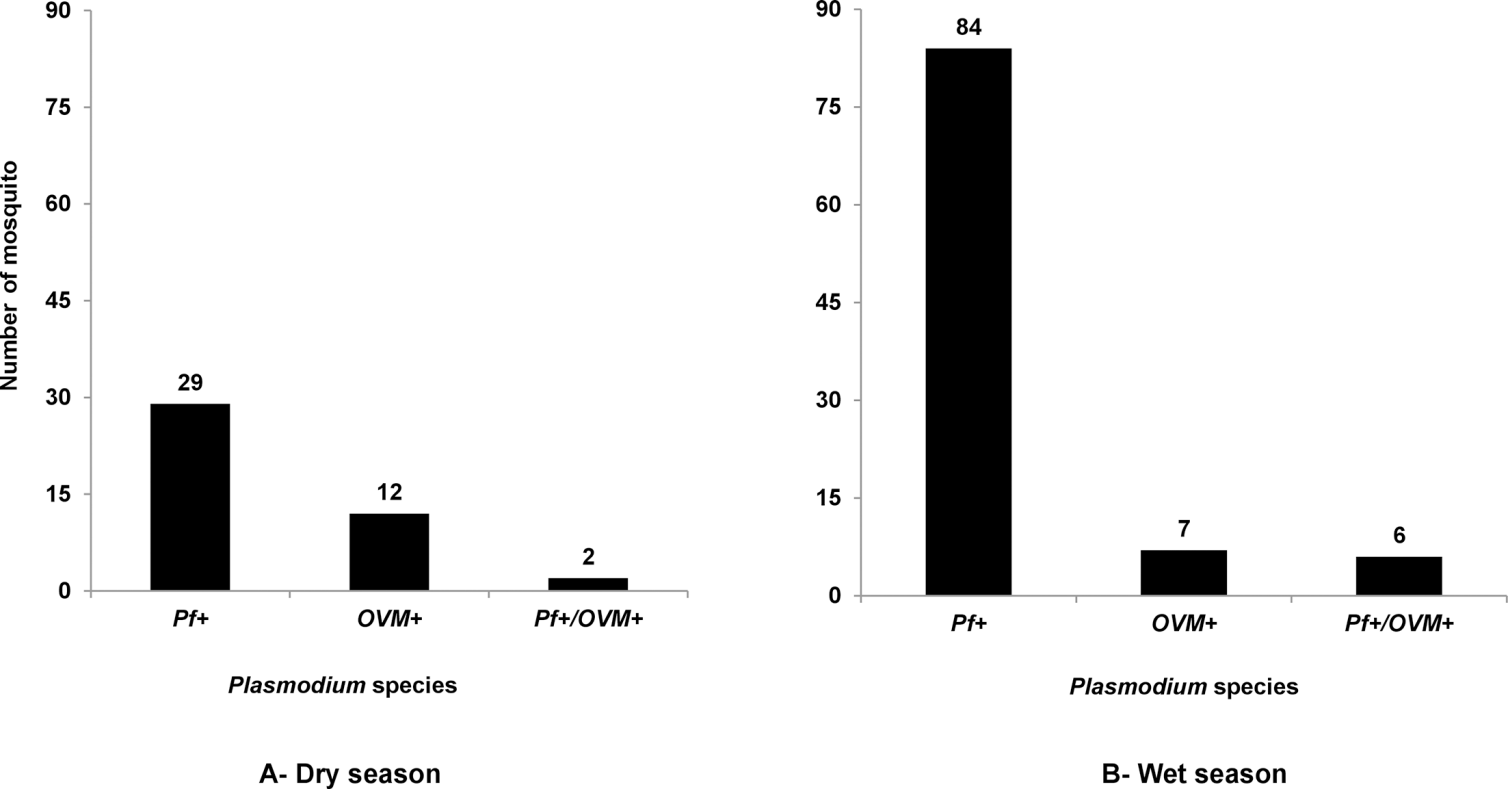
Prevalence of co-infection of *Plasmodium* spp. in mosquitoes *An. gambiae* by real-time PCR. (**A**) The figure shows results of speciation analysis of 43-positive samples of *An. gambiae* collected during the dry season by qPCR. Of the 43-positive samples, 29 were infected by *P. falciparum* (*Pf*+) only (67.6%), 12 samples showed mixed infection with *P. ovale, vivax,* and *malariae* (*P. OVM*) (28%), and a mixed infection with *P. falciparum* and *P. OVM* was observed in 2 samples (2.4%). (**B**) The figure shows results of analysis of 97-positive samples of *An. gambiae* collected during the wet season by qPCR. Among the 97-positive samples, mono-infection with *P. falciparum* was found in 84 samples (86.6%), 7 samples showed mixed infection *P. OVM* (7.3%), and a mixed infection with *P. falciparum* and *P. OVM* was observed in 6 samples (6.1%).

Real-time PCR analysis for detection of *Plasmodium* infection in 554 *An. gambiae* mosquitoes revealed 140-positive samples (43 and 97 mosquitoes, respectively, for the dry and wet seasons) and 414-negative samples (Fig. 1). The overall proportion of *Plasmodium* spp. infection was 25.3% with a variation across the seasons (19% in the dry season and 29% in the wet season) (Table 1). The speciation by real-time PCR of 140 PCR-positive mosquitoes revealed the presence of mono-infection with only *P. falciparum* in 113 (20.7%) mosquitoes (29 for the dry season and 84 for the wet season), and mixed infections with multiple *Plasmodium* species were detected in eight samples (two for the dry season and six for the wet season). Other *Plasmodium* species (*Ovale*, *Vivax,* and *Malariae*) were identified in 19 samples (12 for the dry season and 7 in the wet season). The microbiota diversity of *Anopheles* mosquitoes infected only by *Plasmodium falciparum* (positive) and those uninfected (negative) in this locality was further characterized.

**TABLE 1:** Prevalence of *Plasmodium falciparum* in mosquitoes *Anopheles gambiae* according to the seasons

Season	No. tested	No. infected	No. uninfected	Infection rate (%)
Dry	220	43	177	19
Wet	334	97	237	29
Total	554	140	414	25.3

### Sample characteristics, 16S rRNA sequencing reads, and quality-control statistics

Among the 120 samples sequenced, 118 were selected for downstream analysis due to the high number of quality sequences obtained after quality control. After quality filtering, 1,662,667 reads were assigned to amplicon sequence variants (ASVs) at 99% identity, with an average of 19,832 reads per mosquito sample (Table S1). To determine whether alpha and beta diversity differed across infected and uninfected mosquitoes by season, all samples were rarefied to a depth of 1,000 ASVs per sample. This depth was sufficient to capture the typically low microbiota diversity in individual mosquitoes (Fig. S1).

### Microbiota composition differed between *Plasmodium falciparum* infection status in *Anopheles gambiae*

**Fig 2:**
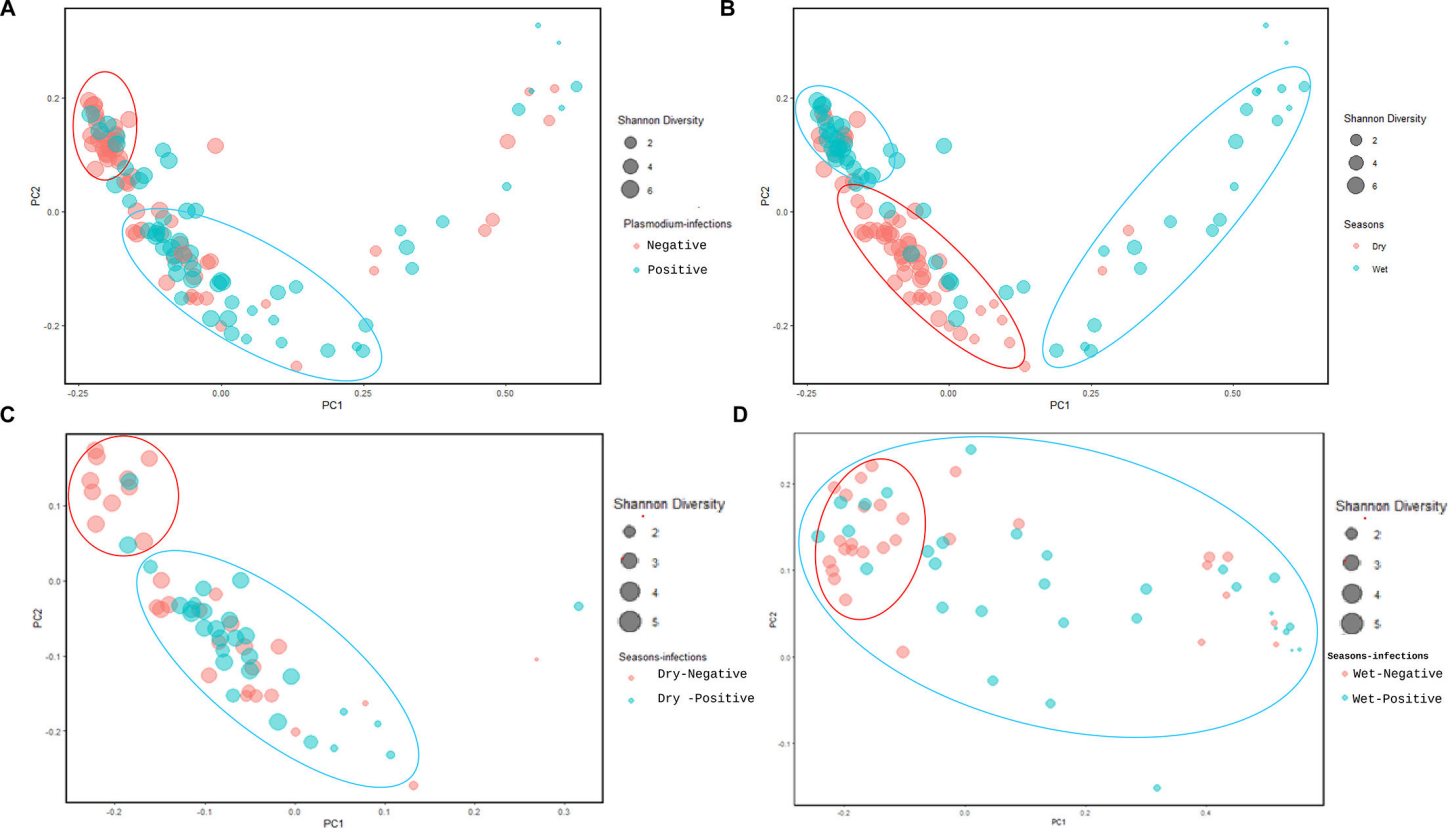
A PCoA plot of Bray-Curtis distances between the microbiota of *Anopheles* infected and uninfected by *P. falciparum* according to the dry and wet seasons. Each point in the plot represents the microbial composition of a single mosquito. (**A**) Bray-Curtis analysis using PERMANOVA (999 permutations) revealed a significant difference in microbial composition between infected and uninfected samples (Pseudo-F = 1.58; *q*-value = 0.04). Red ovals represent uninfected *Anopheles*, while blue ovals represent infected mosquitoes. (**B**) Significant differences in microbial composition were observed between *Anopheles* collected during the dry and wet seasons (Pseudo-F = 9.03; *q* = 0.001). Red ovals represent the dry season, while blue ovals represent the wet season. (**C**) During the dry season, microbial composition significantly differed between infected and uninfected mosquitoes (Pseudo-F = 2.83; *q*-value = 0.01), with red ovals representing uninfected *Anopheles* and blue ovals representing infected mosquitoes. (**D**) In the wet season, significant differences in microbial composition were also observed between infected and uninfected mosquitoes (Pseudo-F = 2.83; *q*-value = 0.01), with red ovals indicating uninfected *Anopheles* and blue ovals indicating infected mosquitoes

Similarities and differences in the microbial composition between *Plasmodium* infection status in *An. gambiae* were analyzed using Bray-Curtis matrices. Bacterial communities varied significantly between infection status, with a clear separation of *Plasmodium*-infected and *Plasmodium*-uninfected samples on the principal coordinates analysis (PCoA) plot (permutational multivariate analysis of variance [PERMANOVA] comparisons of beta diversity, Pseudo-F = 1.58; *q*-value = 0.04, Fig. 2A; Table S2). A PCoA plot of Bray-Curtis distances between the wet and dry seasons also showed a significant difference in bacterial composition (PERMANOVA comparisons of beta diversity, Pseudo-F = 9.03; *q*-value = 0.001; Fig. 2B; Table S2). Based on this highly significant seasonal difference, the microbial composition between *Plasmodium* infection status in *An. gambiae* was further visualized by infection status stratified by season. Bacterial communities varied significantly between infection status, with a clear separation of *Plasmodium*-infected and *Plasmodium*-uninfected samples illustrated by PCoA plot during the dry season (PERMANOVA comparisons of beta diversity, Pseudo-F = 2.83; *q*-value = 0.001, Fig. 2C; Table S2) and the wet season (PERMANOVA comparisons of beta diversity, Pseudo-F = 1.65; *q*-value = 0.06, Fig. 2D; Table S2).

**Fig 3:**
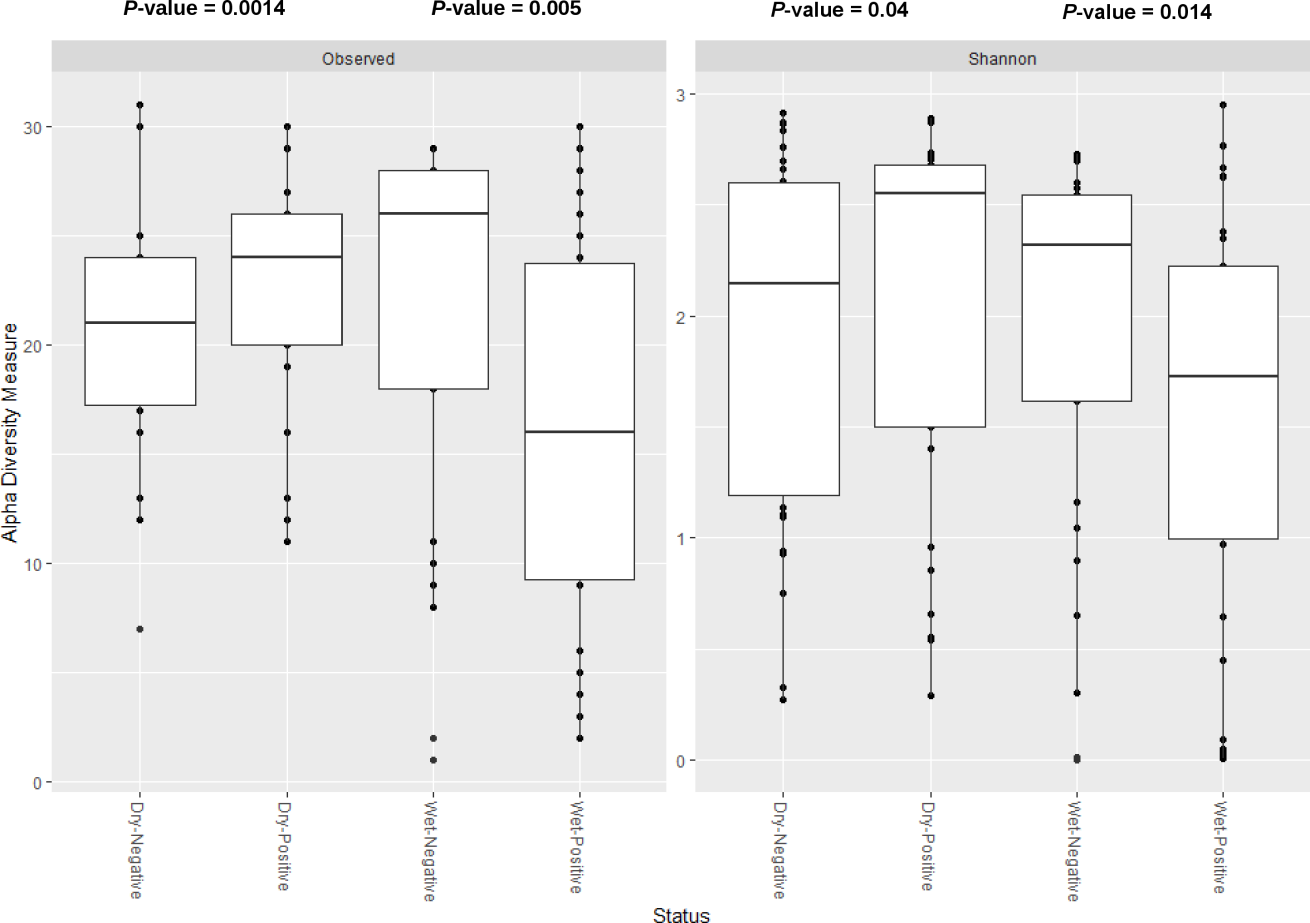
Observed ASVs and Shannon indices showed a significant difference in diversity of bacterial taxa between positive and negative *An. gambiae* from Bankeng, with highest diversity in the negative samples and positive samples, respectively, during the wet and dry seasons. The two *P*-values shown in the figures correspond to comparisons made within each season: one for dry positive vs dry negative and another for wet positive vs wet negative for both indices. These comparisons were performed to assess the effect of *Plasmodium* infection status within each seasonal context. The comparison was performed using the Kruskal-Wallis test. Significance was determined at *P* < 0.05.

Considering microbial diversity within each group, a Kruskal-Wallis test showed no significant difference in observed ASVs (*H* = 0.43, *P* = 0.5) and Shannon index (*H* = 0.47, *P* = 0.49) between *Plasmodium*-infected and *Plasmodium*-uninfected samples for the overall *Anopheles* samples (Fig. S2). However, when stratified by season, significant differences in observed ASVs and Shannon index of microbiota were observed between the *Plasmodium*-infected and uninfected samples, with the highest diversity in the uninfected samples in the wet season and the highest diversity in the infected samples during both the wet and dry seasons (Fig. 3; Table S3).

### Variation in microbial taxa in *Anopheles gambiae* is associated with *Plasmodium falciparum* infection status

The relative proportion of bacteria at different taxonomic levels was further analyzed in both mosquito statuses (infected and non-infected) across the dry and wet seasons, at both phylum and genus levels. A total of 11 bacterial phyla and 100 genera were identified across all samples (Table S4). Following taxonomic analysis of ASVs to the phylum level, 11 phyla were recovered, but only 6 of the 11 ASVs had an overall abundance equal to or greater than 0.1%. The bacterial community was dominated by *Proteobacteria* (64.8%), followed by *Actinobacteria* (15.4%), *Firmicutes* (7.8%), *Deinococcus-Thermus* (3.9%), and *Bacteroidetes* (3.6%) across all samples (Fig. S3).

At the genus level, 99 genera were recovered, but only 46 of the 99 ASVs had an overall abundance equal to or greater than 0.1%. The most abundant genera were *Klebsiella* (16.15%), *Asaia* (14.66%), *Pseudomonas* (12.47%), *Serratia* (12.29%), *Acinetobacter* (6.51%), *Enterobacter* (5.55%), and *Pantoea* (4.42%) across all the samples (Fig. S4; Table S4). The bacterial communities in the mosquitoes differed in composition and abundance between the infected and uninfected groups, as well as across the seasons (dry and wet). The shifts in microbial composition at the phylum and genus levels are shown in Table 2.

**TABLE 2:** Summary of bacterial genus abundance for both mosquito statuses (infected and non-infected) according to the seasons (dry and wet) of all mosquito species

Bacteria	Dry negative (%)	Dry positive (%)	Wet negative (%)	Wet positive (%)	Total abundance (%)
Phylum					
*Proteobacteria*	50.65	57.10	70.06	81.40	64.8
*Actinobacteria*	18.98	16.26	16.22	9.83	15.4
*Firmicutes*	8.62	10.17	7.81	4.48	7.8
*Deinococcus*	8.96	6.27	0.34	0.37	3.9
*Bacteroidetes*	4.07	5.51	2.18	2.32	3.6
Genus					
*Klebsiella*	5.99	9.91	11.01	18.42	16.15
*Asaia*	12.23	13.47	7.74	8.76	14.66
*Pseudomonas*	7.56	8.54	8.98	10.46	12.47
*Serratia*	0.04	5.32	17.84	12.85	12.29
*Acinetobacter*	6.16	7.61	11.16	11.10	6.51
*Enterobacteria*	2.88	3.13	6.25	4.56	5.55
*Pantoea*	0.00	0.05	2.59	8.24	4.42

**Fig 4:**
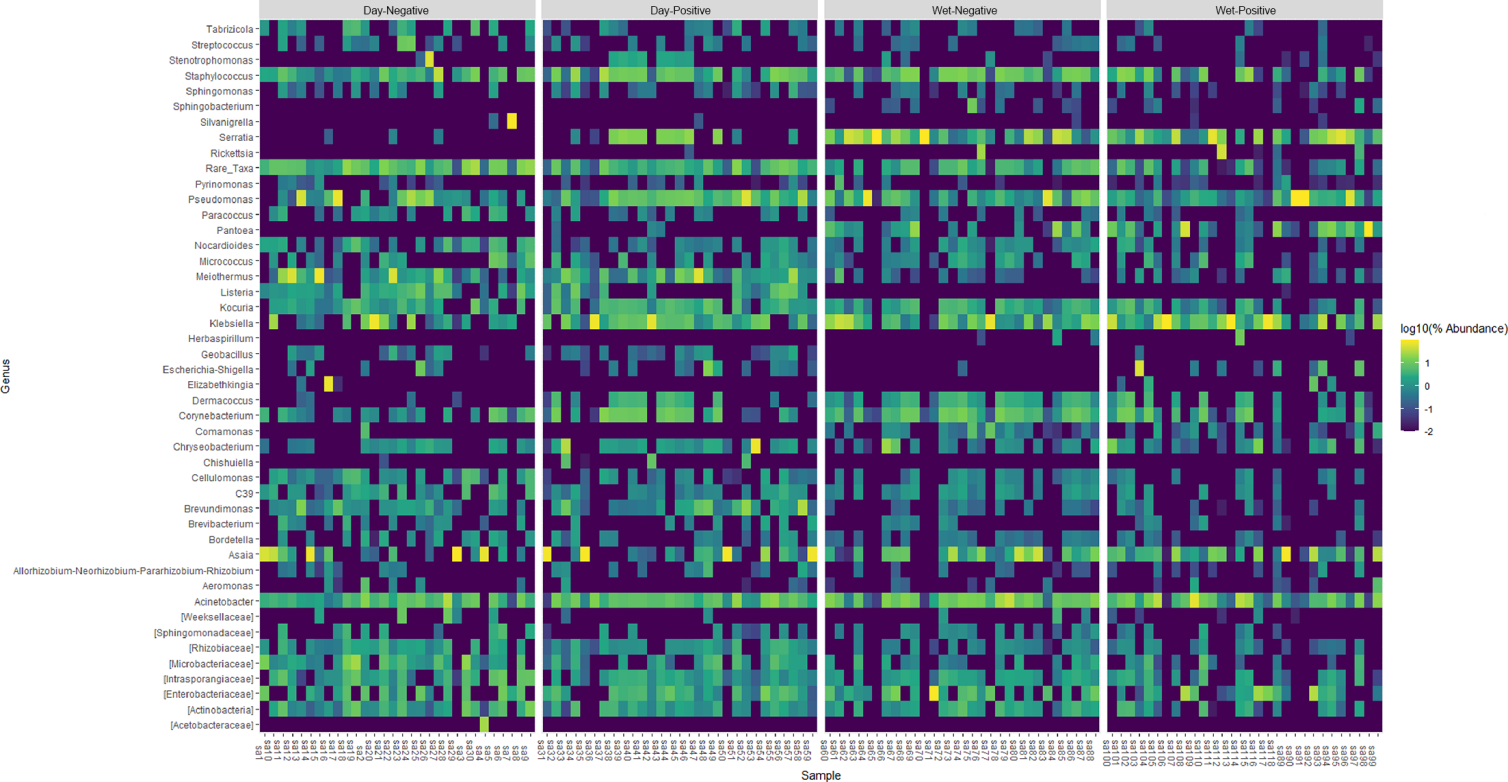
Heatmap showing frequency of annotated ASVs according to the infected status and seasons. Frequency of ASVs from the microbiota of individual dry positive, dry negative, wet positive, and wet negative from Bankeng. The annotation of ASVs was done to the genus level or lowest possible taxonomic level.

In addition, visualizing the data for each sample in order to evaluate any inter-individual variation at the genus level, the bacterial composition in individual sample from each group of samples was very different (Fig. 4; Table S5). Some bacterial taxa were detected more frequently in *Plasmodium*-infected mosquitoes compared to *Plasmodium-*uninfected mosquitoes according to the season. During the dry season, *Asaia* and *Pseudomonas* were predominant for 4 and 5 *Plasmodium*-non-infected samples, respectively, while *Klebsiella, Asaia*, *Pseudomonas,* and *Enterobacter* were predominant, respectively, for in 6, 5, 8, and 6 *Plasmodium*-infected samples. During the wet season, *Klebsiella, Asaia*, *Pseudomonas*, *Serratia*, *Acinetobacter*, and *Pantoea* were predominant, respectively, for 9, 6, 7, 11, 12, and 3 in *Plasmodium-*uninfected samples, while *Klebsiella, Asaia*, *Pseudomonas*, *Serratia*, *Acinetobacter*, *Enterobacter,* and *Pantoea* were predominant, respectively, for 13, 8, 5, 13, 11, 5, and 6 in *Plasmodium-*infected samples (Fig. 4; Table S5).

### Common and differentially abundant bacterial taxa in mosquitoes by infection status and season

**Fig 5:**
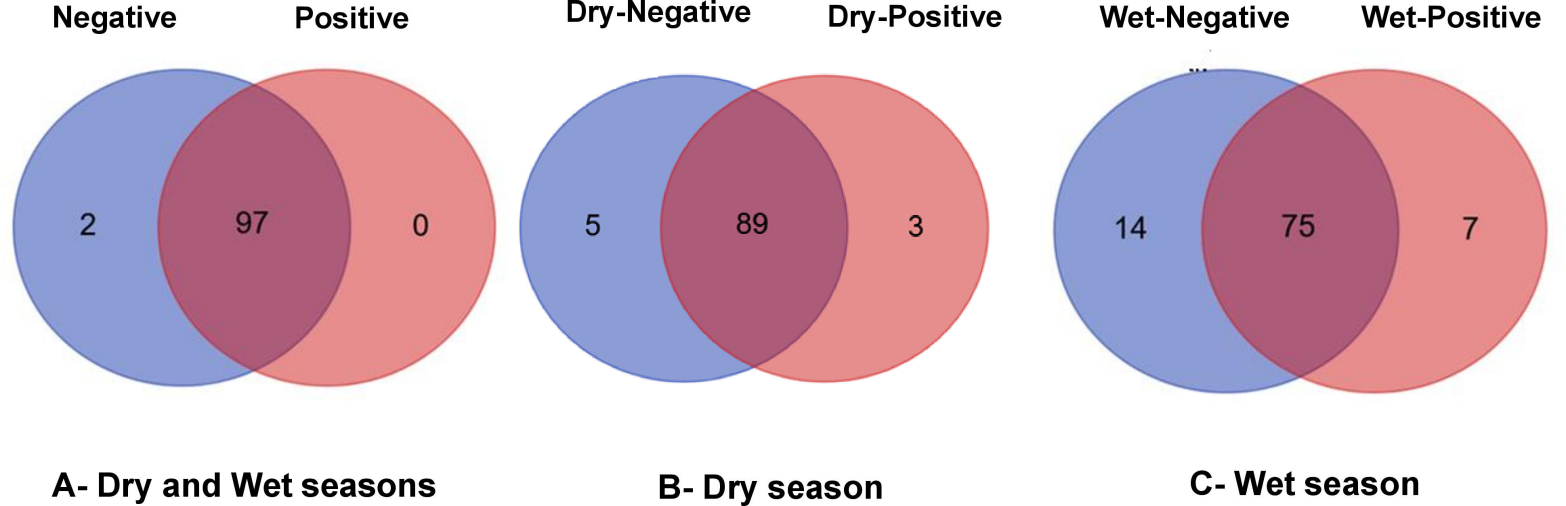
Venn diagrams showing the number of shared or unique bacterial ASVs among infected and uninfected mosquitoes annotated to the genus level. (**A**) Number of unique and shared microbial taxa between infected and uninfected mosquitoes. (**B**) Number of unique and shared microbial taxa between infected and uninfected mosquitoes during the dry season. (**C**) Number of unique and shared microbial taxa between infected and uninfected mosquitoes during the wet season.

Some bacterial genera were specific to one group or shared between groups (Fig. 5; Table S6). Considering all the infected and uninfected mosquitoes without taking into account the season, a total of 99 bacterial genera were identified, 97 of which were shared between the infected and uninfected mosquitoes, while two were unique to uninfected (*Acetobacteraceae*, *Enterococcus*), and no bacteria were unique to infected mosquitoes (Fig. 5A). During the dry season, a total of 97 bacterial genera were identified, 89 of which were shared between the infected and uninfected mosquitoes, while five were unique to uninfected (*Acetobacteraceae*, *Enterococcus*, *Elizabethkingia*, *Comamonas*, *Gemmataceae*), and three (*Aerococcus*, *Pantoea*, *Rickettsia*) were unique to infected mosquitoes (Fig. 5B). During the wet season, a total of 96 bacterial genera were identified, 75 of which were shared between the infected and uninfected mosquitoes, while 14 were unique to uninfected (*Sandaracinaceae*, *Rubellimicrobium*, *Altererythrobacter Hydrogenophaga*, *Nocardiaceae*, *Enterococcus*, *Chamaesiphon PCC-7430*, *Oligoflexales*, *Polaromonas*, *Emticicia, Calothrix PCC-6303*, *Polynucleobacter LB3-76*, *Corynebacterium*), and seven (*Methylophilaceae*, *Geobacillus Elizabethkingia Listeria Gemmataceae, Bacillus*, *Saccharimonadales*) were unique to infected mosquitoes (Fig. 5C).

**Fig 6:**
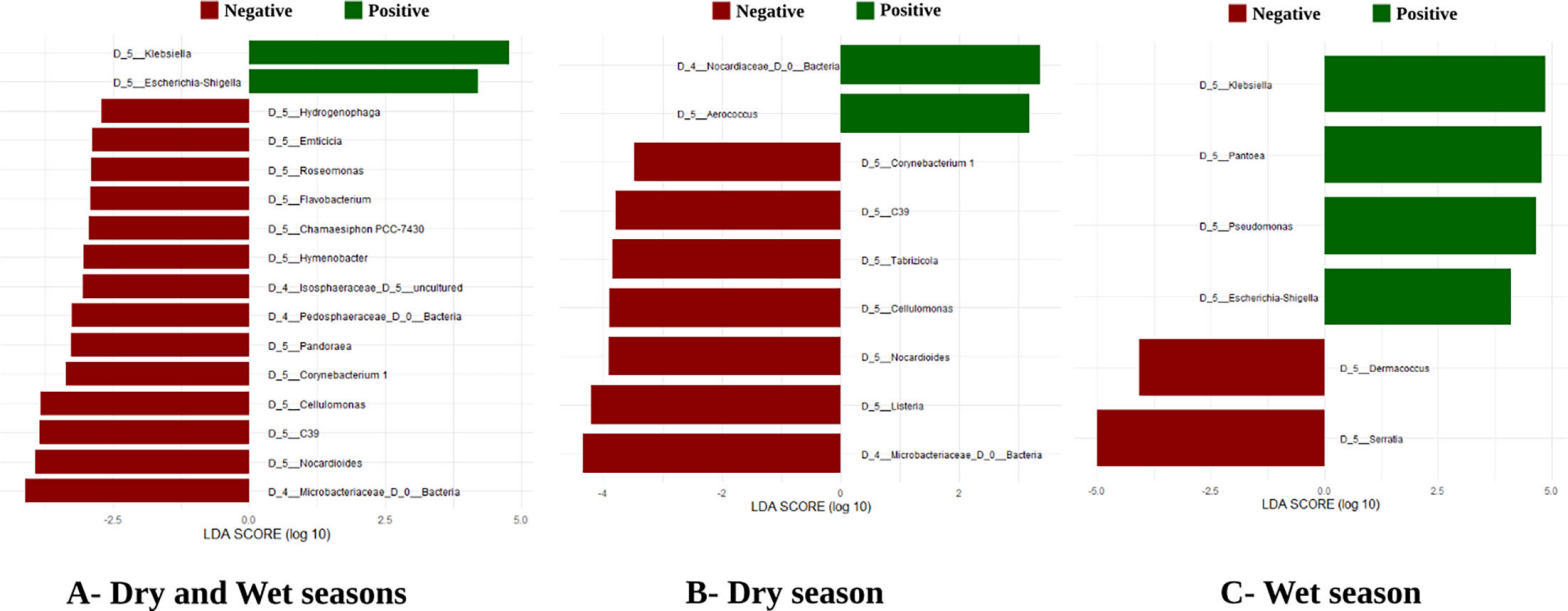
Differentially abundant bacterial genera between infected and uninfected mosquitoes. The red and the green bars represent taxa that were significantly more abundant in the uninfected and infected samples, respectively, at log 10 transformation. Taxonomic levels are designated as genus level. (**A**) Differentially abundant bacterial genera between infected and uninfected for overall mosquitoes. (**B**) Differentially abundant bacterial genera between infected and uninfected mosquitoes during the dry season. (**C**) Differentially abundant bacterial genera between infected and uninfected during the wet season.

Linear discriminant analysis effect size (LEfSe) revealed significant differences in microbiota composition between infected and uninfected mosquitoes. Focusing on the genus level, LEfSe identified 14 bacterial genera as more abundant in uninfected mosquitoes. In contrast, only two bacterial genera were found to be more abundant in infected mosquitoes (Fig. 6A). Linear discriminant analysis effect size (LEfSe) also revealed a highly significant difference in microbiota composition between the dry and wet seasons (Fig. S5). During the dry season, nine bacterial genera were found to be differentially abundant between uninfected and infected mosquitoes, with seven more abundant in uninfected mosquitoes and two more abundant in infected mosquitoes (Fig. 6B). During the wet season, six bacterial genera were differentially abundant, with two more abundant in uninfected mosquitoes and four more abundant in infected mosquitoes (Fig. 6C). Overall, five differentially abundant bacterial taxa at the genus level were unique to infected mosquitoes: *Hydrogenophaga*, *Nocardiaceae*, *Emticicia*, *Corynebacterium*, and *Chamaesiphon PCC-7430*, while three genera (*Aerococcus*, *Pantoea*, and *Elizabethkingia*) were only detected in infected mosquitoes.

## DISCUSSION

The use of microbes is a promising approach to inhibit the development of *Plasmodium* within the mosquito (18). However, comprehensive data on specific bacteria with potential antagonistic effects on malaria parasites remain limited. The objective of this study was to characterize the microbiota composition of *An. gambiae* mosquitoes, both infected and uninfected with *P. falciparum*, and to assess whether seasonality influences this relationship. We found that *P. falciparum* infection profoundly influences the microbiota, but season had minimal impact.

The characterization of the mosquito microbiota and its variability in the presence of natural *Plasmodium* infection represents the next step toward understanding the impact of the microbiota on parasite development within the mosquito midgut. The results revealed diverse bacterial taxa in individual *An. gambiae* samples, most of which had previously been identified in this species in Cameroon (6). Microbiota composition and structure (β-diversity) significantly differed between *Plasmodium*-infected and uninfected *An. gambiae*, with Bray-Curtis distance analysis showing clear separation. This difference could be attributed to certain microorganisms that modulate the immune response of female mosquitoes, thereby significantly influencing their vector competence (19). This difference may also be explained by the presence of specific microbial communities associated with *P. falciparum*-infected and uninfected mosquitoes, as well as seasonal variation in the microbiota (6). More recently, a study found that the composition of the malaria vector microbiota was strongly influenced by seasonality, as well as by factors such as larval breeding sites and/or diet (6, 20). Another study hypothesized that larval microbial exposure, particularly the presence of certain bacterial communities, could impact the vectorial competence of mosquitoes for *Plasmodium* (21). A similar observation was reported by Bassene et al. who conducted a comparative analysis of bacterial diversity in *An. gambiae* and *An. funestus*. The study showed that bacterial microbiota significantly differed between *Plasmodium*-infected and *Plasmodium*-uninfected groups, regardless of species, but was similar among individuals of the same infection status within a species (17). Finally, a comparable pattern of β-diversity variation, driven by some microbes, was also observed in other arthropods including ticks infected and uninfected by *Rickettsia* (22), tsetse flies carrying or free of *Trypanosoma* (23), *Aedes* mosquitoes exposed or not to Zika virus (24), and blackflies harboring or lacking *Onchocerca volvulus* (25).

Microbial diversity within each group showed significant differences in alpha diversity (observed ASVs and Shannon index) between *Plasmodium*-infected and *Plasmodium*-uninfected mosquitoes, with higher diversity in uninfected samples during the wet season and in infected samples during the dry season. This suggests that bacterial diversity in field-collected mosquitoes may be influenced by exposure to the natural environment. During the dry season, the Shannon and observed diversity indices showed significantly higher diversity in *Plasmodium*-infected mosquitoes compared to uninfected ones. This increased diversity in infected mosquitoes could be explained by the fact that rice farms, which serve as primary larval breeding sites during the dry season, may offer a wider range of microbial sources. In contrast, during the wet season, the abundance of breeding sites may reduce exposure to such diverse microbial communities (6, 26). Moreover, blood meals taken by adult female mosquitoes during *Plasmodium* infection could further contribute to this diversity, as the acquired blood may introduce additional microbial (27). In contrast, during the wet season, the Shannon and observed diversity indices showed significant differences between *Plasmodium*-infected and *Plasmodium*-uninfected mosquitoes, with the highest diversity found in the uninfected samples. This may be due to differences in the breeding sites where mosquitoes acquire bacteria. In the rainy season, the number of breeding sites increases due to the presence of both temporary (e.g., standing water) and permanent (e.g., rice fields) breeding sites, which are known to be suitable for *An. gambiae* mosquitoes.

We were also interested in identifying bacterial taxa common and unique to both *Plasmodium*-infected and *Plasmodium*-uninfected mosquitoes and exploring their correlations. We observed that *Plasmodium* infection status is associated with microbiota composition, suggesting a bidirectional interaction where infection could promote certain bacterial taxa, or the microbiota could influence *Plasmodium* infection. Some bacterial genera were either specific to one group or shared between *Plasmodium*-infected and *Plasmodium*-uninfected mosquitoes. Two genera, *Acetobacteraceae* and *Enterococcus*, were unique to uninfected mosquitoes, while none were exclusive to infected mosquitoes. Recently, bacteria associated with *Anopheles* mosquitoes have attracted increasing attention for their potential role in inhibiting *Plasmodium* infection. *Acetobacteraceae* has been detected in wild-caught mosquitoes, indicating its prevalent mutualistic, commensal, or pathogenic association with mosquitoes (28). *Enterococcus* spp. are able to produce toxic molecules with potential antiparasitic activity (such as prodigiosin [29]) that were shown to be toxic to *P. falciparum* (30) and to *Trypanosoma* infection (31, 32). It would be interesting to explore the relationship between these two bacteria and *Plasmodium* infection.

We also aimed to correlate microbes that were differentially abundant between *Plasmodium*-infected and *Plasmodium*-uninfected mosquitoes. Fourteen bacterial genera were significantly more abundant in uninfected mosquitoes, while only two genera were more abundant in infected mosquitoes. These findings align with previous observations showing that several bacterial species are negatively correlated with *P. falciparum* infection. Notably, several identified genera from the *Proteobacteria* phylum may potentially hinder the development of *Plasmodium* in *Anopheles* mosquitoes. The microbiota identified in both infected and uninfected mosquitoes should be the focus of further experimental studies to explore their functions and roles in mosquito fitness. Bacterial residents can contribute to the hosts' fitness by influencing their development (33, 34), reproduction (35), and nutrition (36) with those present in a wide range of taxa potentially involved in their basic functions (37). Little is currently known about the functional roles and interactions of bacteria within mosquito hosts, but several microbes identified in this study have been previously implicated in blood and nectar assimilation (e.g., *Corynebacterium*, *Serratia*), act as an attractant to gravid females (e.g., *Enterobacter*, *Acinetobacter*), or have the ability to impact reproduction (e.g., *Stenotrophom*onas) (2, 38, 39). Additionally, it has been found that the presence of a particular bacteria (e.g., *Serratia*, *Pantoea*, *Klebsiella*, *Pseudomonas, Enterobacter*) can inhibit or promote infection with *Plasmodium* (40–42). Exploration of the relationship between mosquito bacterial communities and intraspecific disease competence would be of value along with work targeting the ecological factors that underpin the relevant variability in their microbiota. Additional studies are needed to validate our results and explore how the full diversity of the *An. gambiae* microbiota could be harnessed for vector control.

Significant differences in microbiota structure and composition were observed between the dry and wet seasons (Fig. S5), with higher abundance in the dry season. This finding strongly supports the effect of seasonality on bacterial composition, which aligns with previous studies (6, 20, 43). Indeed, depending on the season, a significantly more abundant bacteria genera have been identified in uninfected mosquitoes compared to infected mosquitoes. During the dry season, seven bacterial genera were more abundant in uninfected mosquitoes, while two were more abundant in infected mosquitoes. In the wet season, two bacterial genera were more abundant in uninfected mosquitoes, whereas four were more abundant in infected mosquitoes. These results potentially improve the resistance or susceptibility of *Anopheles* mosquitoes to *P. falciparum* infection for some bacterial taxa. This observation was not surprising given the differences that have been noted with *Plasmodium* prevalence infection in this study. The proportion of *Plasmodium* spp. infection was 19% in the dry season and 29% in the wet season. Surprisingly, in the dry season, some bacteria symbionts (*Klebsiella*, *Pseudomonas*, *Pantoea*) known to impact negatively the *Plasmodium* development are more abundant in the infected groups during the wet season, indicating that parasites may interact with the resident microbiota (44). Alternatively, genetic factors, such as allelic polymorphism of immune genes, could regulate the variable levels of the permissiveness of the mosquitoes, as has been previously shown (45). Therefore, it would be of interest to examine the presence/abundance of these bacteria symbionts in wild-caught *Anopheles* mosquitoes, using isolated strains of bacterial (15). The analysis of the microbiota of the whole mosquito, rather than focusing on the digestive system (i.e., salivary glands and midgut), could be considered a limitation of the study’s methodology. However, our decision to analyze whole mosquitoes was guided by several practical and scientific considerations. First, this study was intended as an exploratory, hypothesis-generating analysis aimed at identifying candidate bacterial taxa associated with *Plasmodium* infection. Our findings lay the groundwork for future research that will involve targeted dissections and tissue-specific microbial profiling, particularly of the midgut, to further validate these associations. Second, while the midgut plays a key role in *Plasmodium* development, microbiota present in other mosquito tissues, such as the reproductive tract, may also influence vector competence through systemic immune responses or other mechanisms. Therefore, analyzing the whole mosquito provides a more comprehensive snapshot of the microbial community and its potential associations with infection (46, 47). Finally, the microbes found in specific tissues like the salivary glands or midgut are not necessarily functioning in isolation but may be influenced by or dependent on the microbial communities (or their metabolites) that are located elsewhere (48). Thus, focusing only on specific tissues, like the salivary glands or midgut, would be excluding microbes that are pertinent to vector competence (49, 50).

## MATERIALS AND METHODS

### Study site and mosquito collection

This study was conducted in the locality of Bankeng (center region, 4°40′26.4″N, 12°22′30″E), an irrigated rice-growing village with watercourses along the Sanaga River in a forested area in central Cameroon. Mosquito collections were conducted in this area during the dry season from December 2018 to January 2019 and during the wet season in August 2019. Indoor resting female mosquitoes were collected between 06:00 am and 09:00 am after obtaining verbal consent from the village chief and each household representative. Mosquitoes were collected using Prokopack electrical aspirators (John W. Hook, Gainesville, FL, USA). The collected mosquitoes were kept in individual tubes and then transported to the insectary at the Centre for Research in Infectious Diseases (CRID), Yaoundé, for morphological identification using keys for Afro-tropical anopheline mosquitoes (51). These samples collected were subsequently used to characterize microbiota composition in *Anopheles* infected and non-infected with *P. falciparum* in this locality.

### DNA extraction, molecular identification of *Anopheles* s.l. species, and *Plasmodium* infection

Prior to genomic DNA extraction, individual adult female mosquitoes were surface sterilized by washing them in 70% ethanol for 5 min, followed by two rinses with sterile distilled water to remove superficial bacteria and avoid external contamination. Genomic DNA was then extracted from whole individual mosquitoes using the GeneJET Extraction Kit, following the manufacturer’s recommendations (Thermo Fisher Scientific, based in Waltham, Massachusetts, USA). The isolated DNA was reconstituted in 100 µL of elution buffer, and two aliquots of 50 µL each were prepared and stored at −20°C until further processing. A 50 µL aliquot of the resulting DNA isolate was utilized to build a microbiota library for Illumina sequencing, while the other 50 µL aliquot of the DNA isolate was used for molecular identification of the members of the *An. gambiae* s.l. complex and *Plasmodium* detection. Molecular identification of *Anopheles* was performed using the short-interspersed elements (52).

Detection of *Plasmodium* infection from each whole mosquito was performed using the TaqMan assay as previously described (53). Briefly, two probes were used to determine the presence or absence of *Plasmodium* infection. The first probe, labeled with FAM, detects *P. falciparum*, while the second probe tagged with HEX detects *P. vivax*, *P. ovale,* and/or *P. malariae*.

### 16S rRNA gene amplification, library preparation, and 16S rRNA sequencing

A total of 120 individual *An. gambiae* mosquito samples, categorized by *P. falciparum* infection status, were processed for microbial analysis over two collection seasons. This included 30 randomly selected mosquitoes per infection status and season. Three no-mosquito controls were included throughout the process, from extraction to sequencing, as negative controls, while a mock bacterial community composed of the most frequent bacteria served as a positive control, following the methodology described by Tourlouse et al. (54). Individual DNA samples from these mosquito groups and control samples were sent to Polo d’Innovazione di Genomica, Genetica e Biologia (Infravec), for sequencing using the Illumina MiSeq system. PCR amplification and sequencing procedures targeting the V3-V4 hypervariable region of bacterial and archaeal 16S rRNA gene were performed following the Illumina 16S Amplicon Sequencing Library Preparation Guide (Part # 15044223 Rev. B) using the Nextera XT Index Kit and 16S Amplicon PCR Forward Primer 5'-TCGTCGGCAGCGTCAGATGTGTATAAGAGACAGCCTACGGGNGGCWGCAG-3′and 16S Amplicon PCR Reverse Primer 5'-GTCTCGTGGGCTCGGAGATGTGTATAAGAGACAGGACTACHVGGGTATCTAATCC-3′ following the protocol recommended by Klindworth et al. (55). Then, the samples were subsequently sequenced using the Illumina V2 chemistry, 2 × 250 bp paired-end run.

### Sequence data processing and generation of ASVs table

Raw sequence data derived from the sequencing process were demultiplexed and transferred into FASTA files for each sample, along with sequencing quality files. Resulting data were processed and analyzed using the Quantitative Insights Into Microbial Ecology (QIIME2 v. 2020.2) pipeline as follows. Primers and adapter sequences were removed using the QIIME2 v.2020.2 cutadapt plugins v.2020.2 (56). The divisive amplicon denoise algorithm DADA2 (57) plugin in QIIME2 was used to denoise sequence reads; this step filters out noise and corrects errors in marginal sequences, removes chimeric sequences and singletons, merges paired-end reads, and finally dereplicates the resulting sequences, resulting in high-resolution ASVs for downstream analysis. Using the denoise-paired command, the DADA2 options passed were trunc_len_f: 245 and trunc_len_r: 245. The ASVs were further filtered to remove ASVs associated with the negative control, and any reads assigned to PCR control were also filtered using the filtered taxa plugin of QIIME2 2020.2. In addition, ASVs with a minimum frequency of 200 were removed to have more representative reads for all the samples. Supplementary data (Table S1) show sequencing reads and ASV summary statistics.

### Diversity indices

Analysis of microbial diversity was described within (alpha diversity) and between (beta diversity) samples. The Shannon diversity index and observed features alpha diversity indices were calculated to estimate the inter-individual variation of bacterial diversity in different infection status (*Plasmodium* infected or uninfected) stratified by seasons (dry and wet seasons). The observed ASVs metric was used to estimate the number of unique ASVs (or richness) present within each mosquito infection status, while the Shannon diversity index was used to estimate both ASV richness and evenness. To determine whether alpha diversity differs across infection status per season, all samples were rarified to a depth of 1,000 ASVs per sample, which was sufficient to capture the typical low microbiota diversity in individual mosquitoes. The resulting average Shannon and observed indices were compared between *Anopheles* mosquitoes presenting two different infection statuses (*Plasmodium* infected or not) for both seasons (dry and wet) using pairwise Kruskal-Wallis tests with Benjamini-Hochberg false discovery rate (FDR) corrections for multiple comparisons.

Principal components analysis, using the Bray-Curtis dissimilarity index and ordination plots, was performed to determine differences in bacterial communities across samples from different groups. These differences were quantified using permutational multivariate analysis of variance (PERMANOVA). No discernible differences were found between the results of rarefied and non-rarefied data. Therefore, the Bray-Curtis dissimilarity indices based on rarefied data were visualized using PCoA plots in R (58) and non-metric multidimensional scaling using the phyloseq R package (59). Finally, differential abundance testing was conducted to identify potential taxonomic groups that could serve as biomarkers associated with a specific condition, mainly *Anopheles* infectivity with *Plasmodium*. The significance for both pairwise analyses was set to *q* < 0.05 (i.e., post FDR *P*-value corrections).

### Taxonomic analysis of microbial taxa

Taxonomic analysis of ASVs was performed using QIIME2’s pre-trained Naïve Bayes classifier (60) and q2-feature-classifier plugin (61). Prior to analysis, the classifier was trained on the QIIME-compatible 16S SILVA reference (99% identity) database v.128 (62), and using the extract-reads command of the q2-feature-classifier plugin, the reference sequences were trimmed to the V3-V4 region (450 bp) of the 16S rRNA gene. The qiime feature-table heatmap plugin was subsequently used to visualize the resulting relative abundance of annotated ASVs across different groups of samples (dry negative, dry positive, wet negative, and wet positive). The plugin’s metrics and clustering methods were set to Bray-Curtis and features, respectively. Only annotated ASVs with counts  ≥2,000 were included in the heatmaps.

### Testing for differentially abundant microbial features between *P. falciparum*-infected and *P. falciparum*-non-infected mosquitoes

The list of bacterial genera, as derived from ASVs, in each sample and group of samples was compared using Venn diagrams. These Venn diagrams were created using an online tool (http://bioinformatics.psb.ugent.be/webtools/Venn/).

Differentially abundant microbial taxa across mosquitoes with two different infection statuses in both seasons were identified using the linear discriminant analysis (LDA) effect size method (LEfSe) (63). Annotated ASVs were converted into abundance tables and uploaded to LEfSe Galaxy v.1.0 (http://huttenhower.sph.harvard.edu/lefse/). Using default parameters, an alpha value of 0.05 was applied for both the factorial Kruskal-Wallis and pairwise Wilcoxon tests within LEfSe. A threshold of >2 was set on the resulting logarithmic LDA score to identify differentially abundant ASVs. The effect sizes of these ASVs were visualized as bar plots using R software.

## Data Availability

The raw sequencing reads from this study, including those from negative (blank) and positive controls, along with sample metadata, have been deposited in the Sequence Read Archive (SRA) under BioProject PRJNA1231452, with accession numbers SAMN47212096 to SAMN47213749.

## SUPPLEMENTAL MATERIAL

Fig. S1 msphere.00490-25-s0001.tiff Rarefied curve.

Fig. S2 msphere.00490-25-s0002.tiff Alpha diversity between *Plasmodium*-infected and -uninfected samples.

Fig. S3 msphere.00490-25-s0003.tiff Taxonomic analysis at the phylum level.

Fig. S4 msphere.00490-25-s0004.tiff Taxonomic analysis at the genus level.

Fig. S5 msphere.00490-25-s0005.tiff LEfSe microbiota composition between the dry and wet seasons.

Legends msphere.00490-25-s0006.docx Legends for supplemental items.

Tables S1 to S6 msphere.00490-25-s0007.xls Sequence data results.

ASM does not own the copyrights to Supplemental Material that may be linked to, or accessed through, an article. The authors have granted ASM a non-exclusive, world-wide license to publish the Supplemental Material files. Please contact the corresponding author directly for reuse.
