# Supplementary figures and images for: Microbiota diversity of Anopheles gambiae in Bankeng, southern Cameroon, and its association with Plasmodium falciparum infection
Source: mSphere. 2025 Dec 5;10(12):e00490-25. doi: 10.1128/msphere.00490-25 (PMC12724382; doi:10.1128/msphere.00490-25)

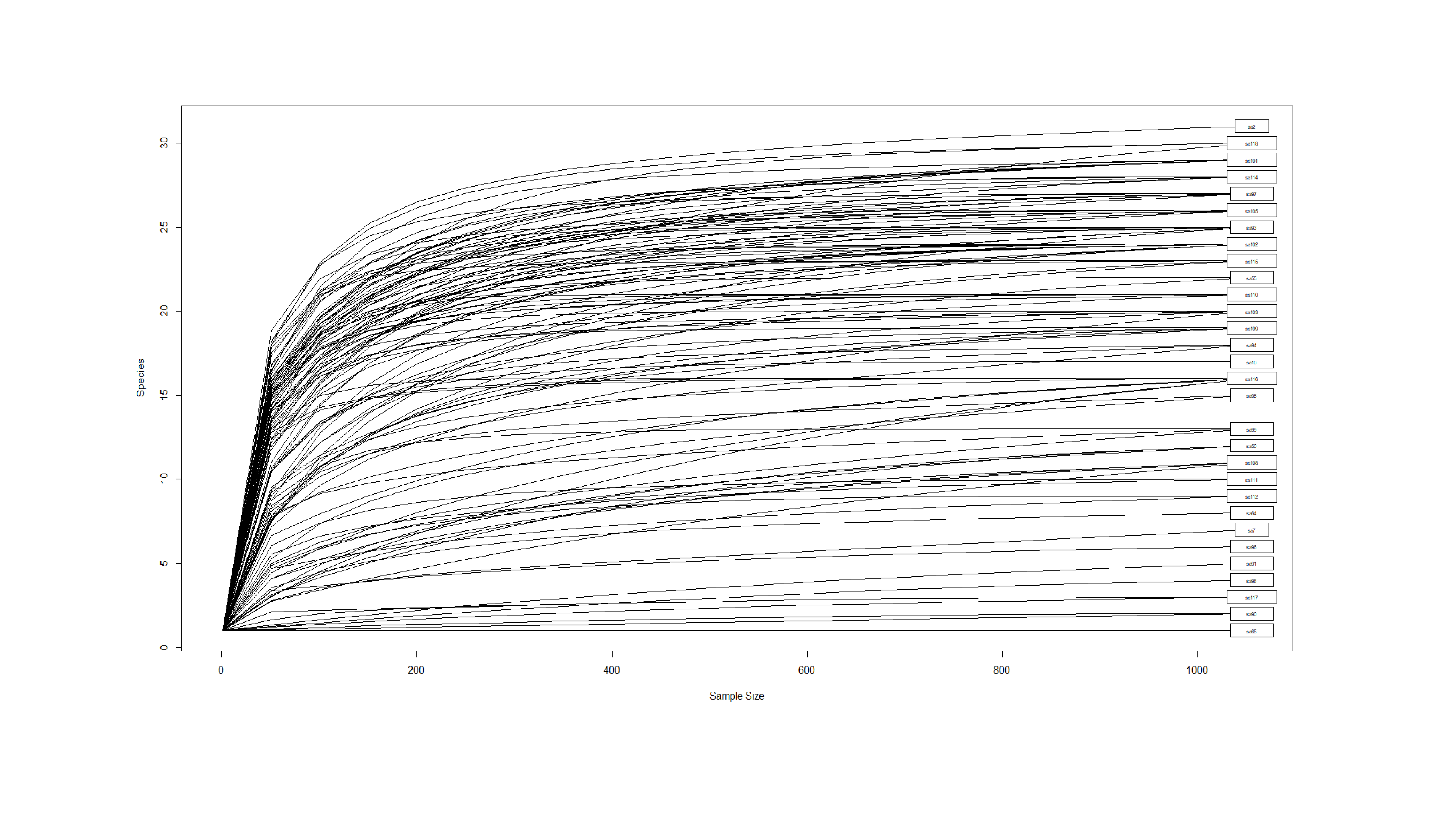

Supplement: Fig. S1 — Rarefied curve. [file msphere.00490-25-s0001.tiff]

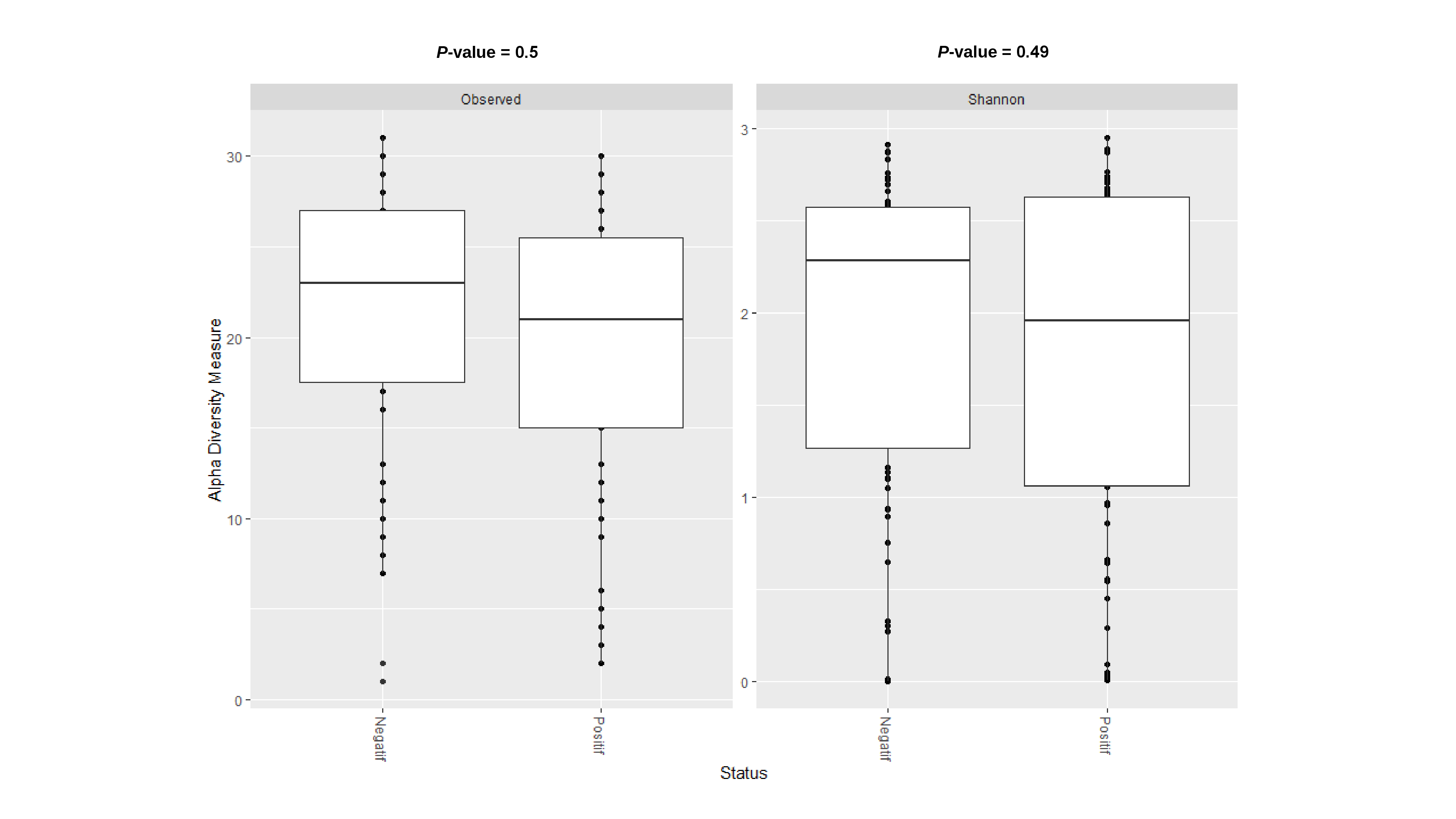

Supplement: Fig. S2 — Alpha diversity between Plasmodium-infected and -uninfected samples. [file msphere.00490-25-s0002.tiff]

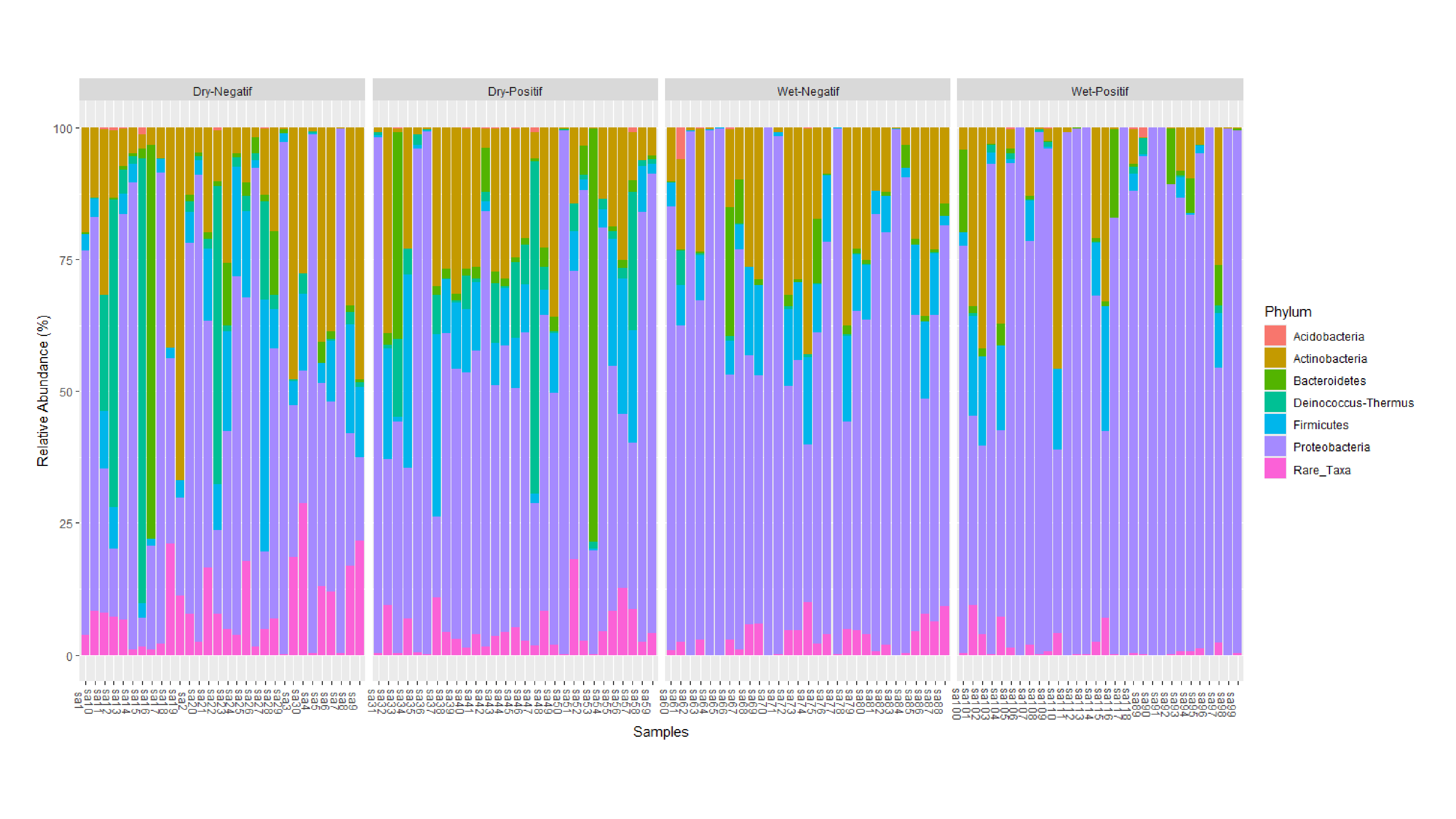

Supplement: Fig. S3 — Taxonomic analysis at the phylum level. [file msphere.00490-25-s0003.tiff]

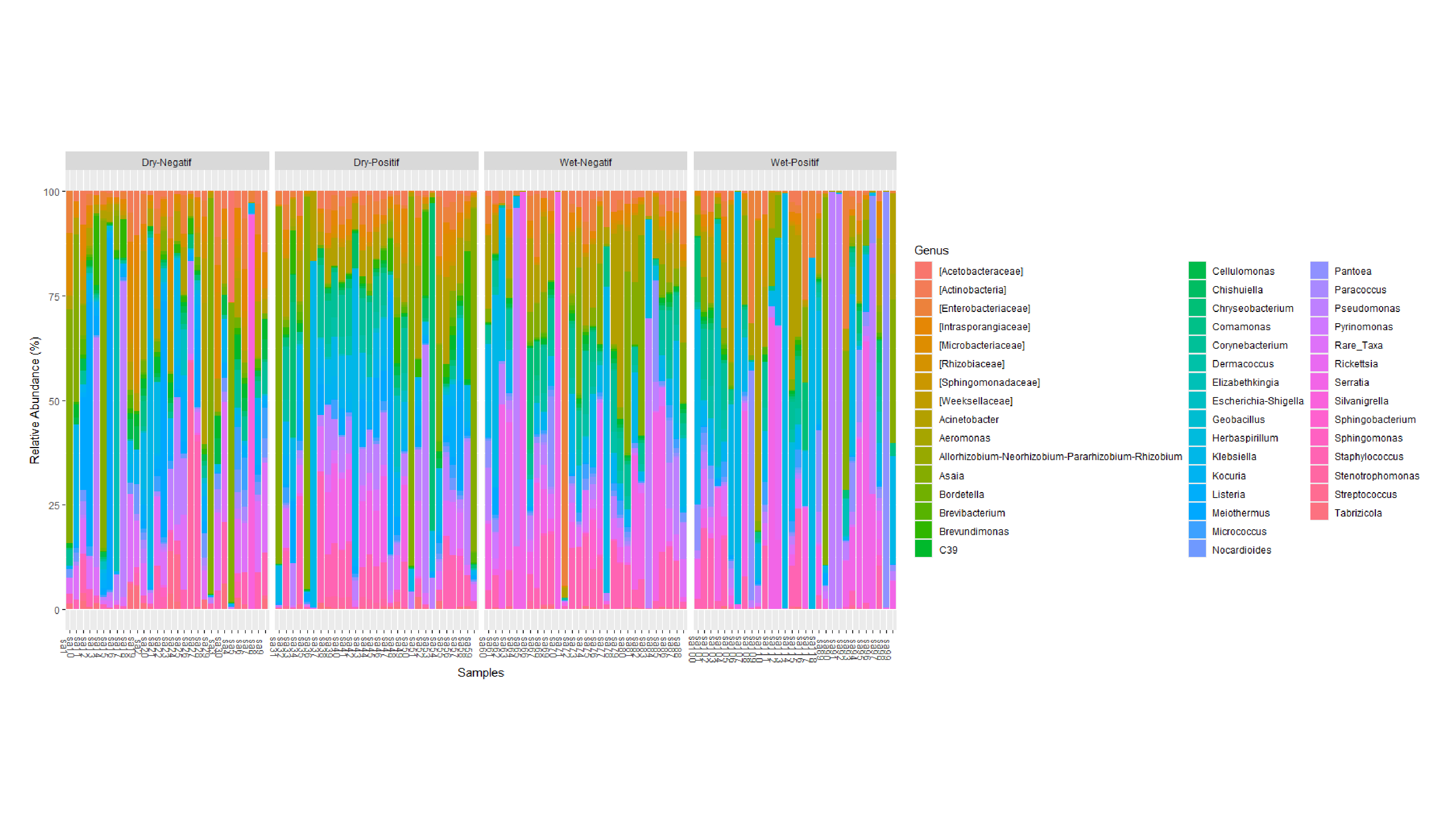

Supplement: Fig. S4 — Taxonomic analysis at the genus level. [file msphere.00490-25-s0004.tiff]

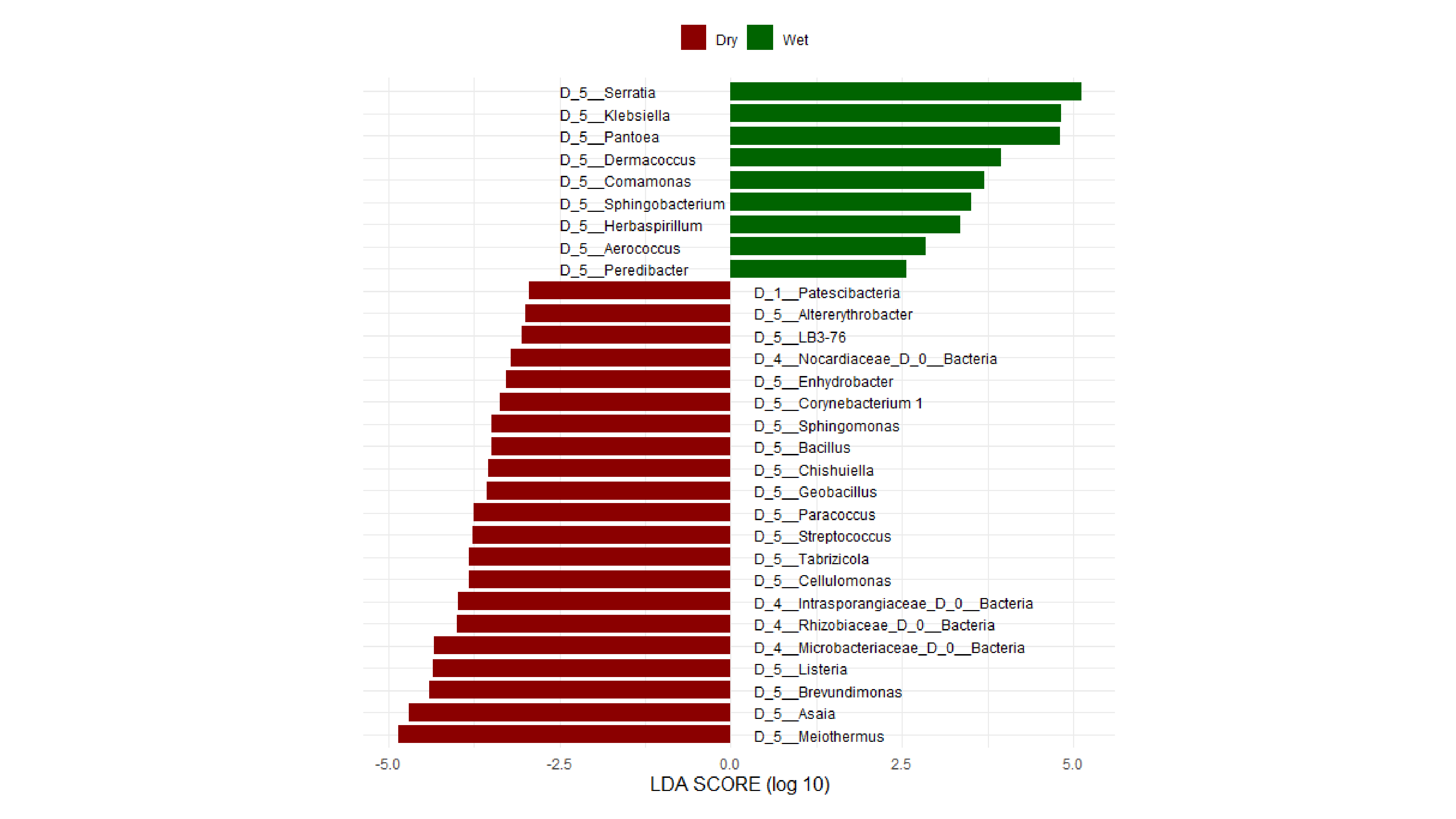

Supplement: Fig. S5 — LEfSe microbiota composition between the dry and wet seasons. [file msphere.00490-25-s0005.tiff]
