# Supplementary material for: Microbiota diversity of Anopheles gambiae in Bankeng, southern Cameroon, and its association with Plasmodium falciparum infection
Source: mSphere. 2025 Dec 5;10(12):e00490-25. doi: 10.1128/msphere.00490-25 (PMC12724382; doi:10.1128/msphere.00490-25)
Supplement: Legends — for supplemental items. [file msphere.00490-25-s0006.docx]

**Supplementary Figures**

**Fig. S1.** Rarefaction curves & depth.

The following figures show rarefaction curves of the alpha diversity index (Observed ASVs and Shannon) for each mosquito tested. All curves plateaued, indicating that additional sampling efforts did not result in changes in abundance and evenness of microbial taxa per sample. At each sampling depth shown, each curve shows the average Observed and Shannon diversity value, along with the range (boxplots-minimum, median and maximum) of values from 10 rarefaction iterations.

**Fig. S2.** Shannon and Observed diversity indices showed no significant difference between infected and uninfected mosquitoes. The comparison was performed using the Kruskal-Wallis test. Significance was determined at p <0.05.

**Fig. S3**. Bar plots showing the relative abundance of taxonomically annotated amplicon sequence variants (ASVs) from adults *An. gambiae.* AVSs showing an overall abundance equal to or greater than 0.1% were taxonomically annotated to the phylum level. The bar plots show the relative abundance of annotated ASVs for individual samples across the infection status during both seasons. In general, microbiota was dominated by *Proteaobacteria* followed by *Actinobacteria*, *Firmicutes* Deinococcus*-Thermus*, and *Bacteroidetes.*

**Fig. S4**. Bar plots showing the relative abundance of taxonomically annotated amplicon sequence variants (ASVs) from adults *An. gambiae.* ASVs showing an overall abundance equal to or greater than 0.1% were taxonomically annotated to the genus level. The bar plots show the relative abundance of annotated ASVs for individual samples across the infection status during both seasons. In general, microbiota was dominated by *Klebsiella* (16.15%), *Asaia* (14.66%), *Pseudomonas* (12.47%), *Serratia* (12.29%), *Acinetobacter* (6.51%), *Enterobacter* (5.55%) and *Pantoea* (4.42%).

**Fig. S5.** Differentially abundant bacterial genera between mosquitoes collected during the dry and the wet season. The red and the green bars represent taxa which were significantly more abundant in the dry and wet samples, respectively, at log 10 transformation. Taxonomic levels are designated as genus level.

**Supplementary Tables**

**Table S1.** Sequencing outputs and proportion of reads used for downstream analysis following quality control and dereplication.

**Table S2.** Beta-diversity comparisons show differential bacterial composition between infections status and between seasons. Pair-wise comparisons of beta diversity (Bray Curtis) of mosquitoes’ microbiota between infection status (Negative vs Positive) in dry season, and in wet season, showed significant differences in bacterial composition. Comparisons were conducted using PERMANOVA (999 permutations) tests with Benjamini-Hochberg FDR correction (q-value). Significance is set to q-value (adjusted p-value).

**Table S3.** Alpha-diversity comparisons show differential bacterial composition between the infections status according to the seasons.

**Table S4.** Taxonomic annotation and frequency of ASVs from *Anopheles gambiae* mosquitoes infected and non-infected by *Plasmodium falciparum* collected during the dry and wet seasons.

**Table S5.** Number of individual samples with predominant bacterial at the genus level

**Table S6.** Summary of taxonomic annotations and frequency of ASVs used to construct the Venn diagram.
